# Research Advances in CKLF-like MARVEL Transmembrane Domain-containing Family in Non-small Cell Lung Cancer

**DOI:** 10.7150/ijbs.33733

**Published:** 2019-09-07

**Authors:** Keheng Wu, Xiaoman Li, Huadi Gu, Qiao Yang, Yingying Liu, Liang Wang

**Affiliations:** 1 China Medical University-The Queen's University of Belfast Joint College, Shenyang, Liaoning, 110122, China; 2 Institute of Translational Medicine, Key Laboratory of Medical Cell Biology, Ministry of Education, China Medical University, Shenyang, Liaoning, 110122, China; 3 Department of Pathology, College of Basic Medical Sciences, China Medical University, Liaoning, 110122, China

**Keywords:** CMTM, CKLFSF, MARVEL, tumor suppressor gene, non-small cell lung cancer

## Abstract

CKLF-like MARVEL transmembrane domain-containing member (CMTM) is a new gene family first cloned and reported in 2001. The CMTM family consists of nine members including CKLF and CMTM1-CMTM8, which are located on different chromosomes. Besides exhibiting extensive chemotactic activity, the CMTM family plays an important role in the hematopoiesis system, the immune system, the cardiovascular system and the male reproductive system. Recent in-depth research has also revealed that CMTM is closely associated with the genesis, development and metastasis of tumors, displaying opposing activities in diverse human tumors. In this review, we discuss the structural and functional characteristics of the CMTM family and summarize latest research findings of the relationship between several CMTM members and non-small cell lung cancer.

## Introduction

Chemokine-like factor superfamily members (*CKLFSF*) are part of a novel gene family that was first described in 2001 1, 2. Han *et al.* cloned a novel cytokine, chemokine-like factor 1 (CKLF1), from the phytohemagglutinin-stimulated leukemia cell line U937, which was used to study interleukin-10 inhibition by suppression subtractive hybridization. They also cloned and validated CKLF-like MARVEL transmembrane domain-containing members (*CMTM*) by reverse transcription PCR 2-4. In 2005, the International Human Genetics Nomenclature Committee renamed CKLFSF1-8 as CMTM1-8 according to their molecular structures 5-7. *CMTM1-8* and chemokine-like factor (*CKLF*), are located on different chromosomes. *CKLF* and *CMTM1-4* are grouped together on chromosome 16q22.1 to form a gene cluster, *CMTM5* is independently located on 14q11.2, and CMTM6-8 are co-located on chromosome 3p23. Their coding products include chemokines and the transmembrane 4 superfamily (TM4SF). CMTM1 in particular shows the most similarity to chemokines, CMTM8 resembles TM4SF, and the biological features of CMTM2-7 are somewhere in between 2, 8-15.

Functionally, CMTMs not only have broad-spectrum chemotactic activity, but also play an important biological role in the hematopoietic, immune, cardiovascular, and male reproductive systems 16-20. Additionally, CMTMs are associated with autoimmune and hematopoietic diseases 21-23, and with the genesis, development, and metastasis of multiple malignancies 24-27. Each CMTM member has different biological functions in different tumors, although they collectively play a suppressive role. Many cancers of the digestive tract such as esophageal, gastric, and oral cancer involve CMTM3 in several different pathways 28-33, while, cancers of digestive glands, like hepatocellular carcinoma, are also associated with CMTM members 34, 35. Moreover, researches in genitourinary cancers indicate that CMTM3 and CMTM8 suppress tumor cell reproduction and migration 36-40.

Isoform CMTM1_v17 of CMTM1, a newly discovered member of the family, functions in breast cancer carcinogenesis and is a potential novel therapeutic target 41. CMTM1 overexpression in the glioblastoma cell line A172 also promotes the proliferation and migration of tumor cells, which is likely to be achieved by the activation of epidermal growth factor receptor (EGFR), Src family kinase, and Wnt signaling 42. However, the specific mechanism of CMTM1 in the development of glioblastoma is unclear. Moreover, although CMTM3 showed anti-cancer effects in testicular, prostate, liver, gastric, and kidney cancers as well as other tumors, it had a cancer-promoting effect in glioma 42. Similarly, CMTM7 showed a cancer-promoting role in glioma but an anti-cancer role in non-small cell lung cancer (NSCLC) 42, 43, while CMTM4, CMTM5, and CMTM8 functioned in tumor suppression in a variety of solid tumors 44-46. Thus, the functions of CMTM family members are diverse in different malignancies, so it is important to further study their roles and mechanisms in tumorigenesis, development, and metastasis. This could identify new molecular targets for tumor detection and gene therapy, bringing new hope for cancer patients. A review of the literatures suggests that several CMTMs are closely involved in the malignant progression of NSCLC, and we discuss their relationship in the current review, with particular focus on CMTM7.

### CMTM1 and NSCLC

**Figure 1:**
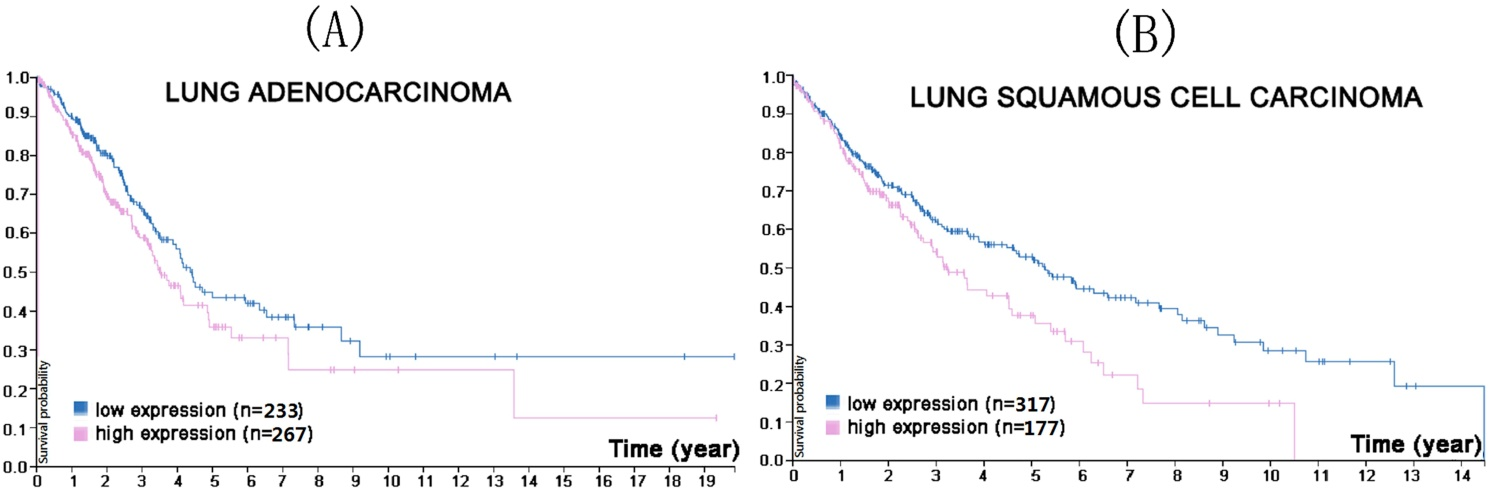
In the course of lung adenocarcinoma (A) and lung squamous cell carcinoma (B) progression, lower CMTM1 expression was associated with a higher survival probability (p<0.053 for lung adenocarcinoma, p<0.013 for lung squamous cell carcinoma). (Image credit: Human Protein Atlas. https://www.proteinatlas.org/ENSG00000089505-CMTM1/pathology/tissue/lung+cancer)

CMTM1 is located on chromosome 16 and encodes at least 23 alternative spliced isoforms (CMTM1_v1-v23) 47. The CMTM1_v1-16 protein is encoded by open reading frame (ORF)1 while ORF2 encodes CMTM1_v17-23 41. CMTM1_v17 consists of 149 amino acids and has clear tissue expression specificity, being highly expressed in the testes and prostate tissues, but low or undetectable in many other tissues 48. Recent studies have shown that CMTM1_v17 is also highly expressed in a variety of solid tumors (breast cancer, kidney cancer, lung cancer, and ovarian cancer) and that it can activate nuclear factor-kappa B (NF-κB) signaling to promote cell proliferation and partially resist apoptosis induced by tumor necrosis factor-α 41. Additionally, CMTM1_v17 expression is strongly linked with chemotherapy resistance and poor prognosis in early stage NSCLC patients who have received neoadjuvant chemotherapy 49. Protein Atlas search found that lower CMTM1 expression was associated with a higher survival probability in both lung squamous cell carcinoma and adenocarcinoma (Figure 1).

### CMTM5 and NSCLC

**Figure 2:**
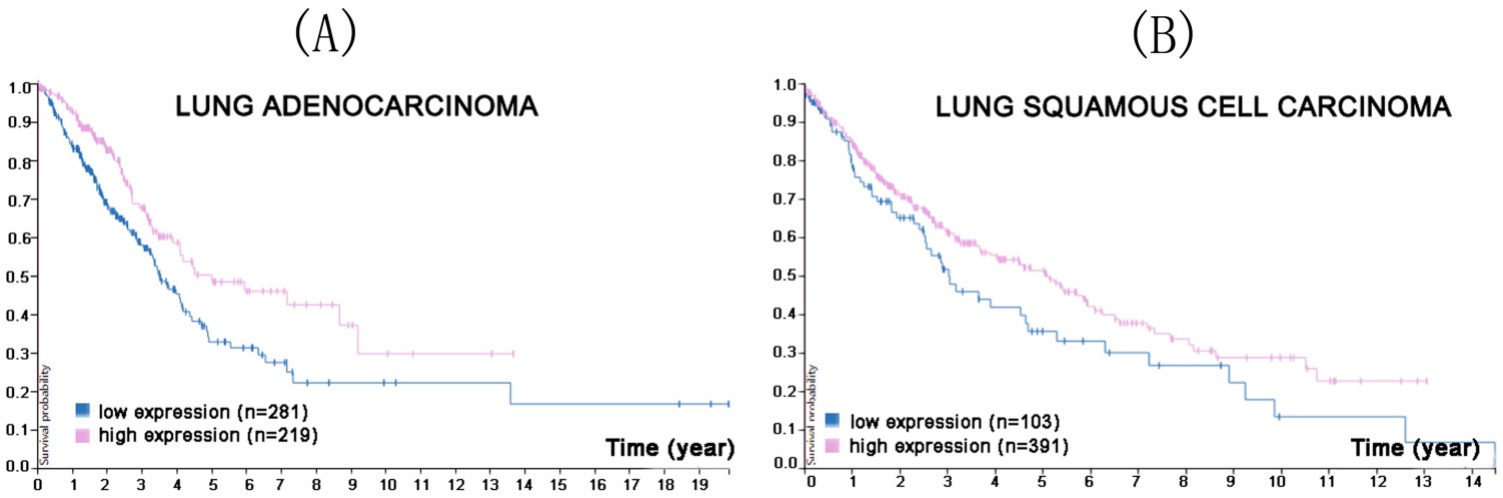
In the course of lung adenocarcinoma (A) and lung squamous cell carcinoma (B) progression, reduced CMTM5 expression was associated with a lower survival probability (p<0.0021 for lung adenocarcinoma, p<0.057 for lung squamous cell carcinoma). (Image credit: Human Protein Atlas. https://www.proteinatlas.org/ENSG00000166091-CMTM5/pathology/tissue/lung+cancer)

*CMTM5* is located at 14q11.2 and does not cluster with any other CMTM members, but is closely linked to the interleukin 25 gene 50. It includes at least six mRNA splicing bodies, CMTM5-v1-v6, of which CMTM5-v1 is the most conserved. CMTM5 is widely expressed in many normal tissues such as brain, prostate and small intestine, and has a secretory form 51, although its expression is greatly reduced in many tumor tissues, such as prostate cancer, cervical cancer, ovarian cancer, leukemia, glioma, and digestive system tumors 52-56. Its anti-cancer mechanism are not clear, but may be related to promoter methylation or the function of multiple signal transduction pathways 11. A nude mouse model of prostate cancer xenografts was established to confirm the inhibitory effect of CMTM5 55. After 3 weeks, CMTM5 adenovirus was injected into the tumor, and CMTM5 was shown to inhibit the growth of prostate cancer by downregulating the expression of vascular endothelia growth factor and NF-κB 51, 55. Another team confirmed that CMTM5 inhibits the proliferation and migration capacity of prostate cancer cells 54. Moreover, a Protein Atlas search found that reduced CMTM5 expression was associated with a lower survival probability in lung adenocarcinoma and squamous cell carcinoma (Figure 2).

### CMTM6 and NSCLC

*CMTM6* is another member of the CMTM superfamily located at 3p22.3 adjacent to *CMTM7*. It is 21600 nucleotides long and encodes three transcripts, CMTM6-001 to CMTM6-003, but only CMTM6-001 with four introns can be successfully be translated. CMTM6 is a 183 amino acid protein with a typical MARVEL domain, and is mainly expressed in the lung, liver, immune organs like the spleen, lymph nodes, and tonsils, as well as reproductive organs 2. CMTM6 also showed clinical importance in hepatocellular carcinoma (HCC) 57. Histological revealed higher CMTM6 expression in HCC tissues than in adjacent normal tissues, and its expression correlates with metastasis, tumor staging and the expression of alpha-fetoprotein. High CMTM6 expression is also associated with poor prognosis in malignant gliomas, which is caused by the inhibition of T-cell-mediated anti-tumor immunity 58. This finding is consistent with the previously discovered identity of CMTM6 as a key regulator of programmed cell death ligand 1 (PD-L1), which functions as an immune checkpoint inhibitor of T cells 26, 59. Mezzadra *et al*. 26 and Burr *et al*. 59 recently reported the regulation of PD-L1 by CMTM6 and its pro-oncogenic role in several cell lines following clustered regularly interspaced palindromic repeats (CRISPR)-CRISPR-associated protein (Cas)9 deletion of *CMTM6* and subsequent decrease in PD-L1 expression. CMTM6 was shown to co-localize with PD-L1 on the plasma membrane and recycling endosomes, aiding its return to the cell surface and impeding its degradation. It enhanced the resistance of PD-L1 to endoglycosidase H, preventing it from undergoing deglycosylation.

**Figure 3:**
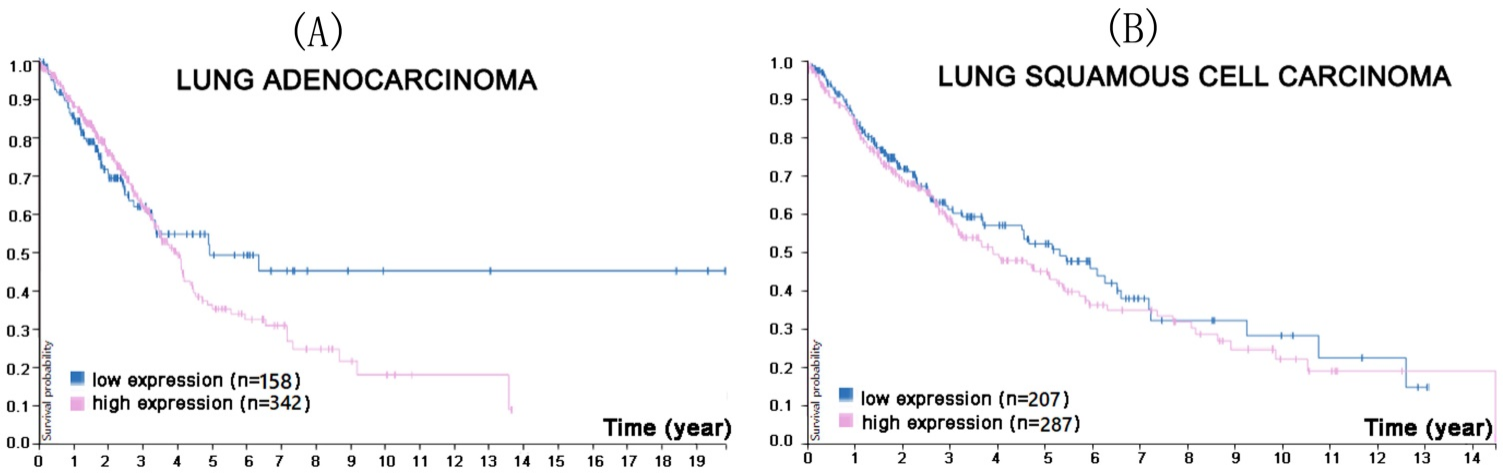
In the course of lung adenocarcinoma (A) and lung squamous cell carcinoma (B) progression, higher CMTM6 expression was associated with a lower survival probability (p<0.4 for lung adenocarcinoma, p<0.33 for lung squamous cell carcinoma). (Image credit: Human Protein Atlas. https://www.proteinatlas.org/ENSG00000091317-CMTM6/pathology/tissue/lung+cancer)

Mezzadra also transfected the NSCLC cell line H2030 with short hairpin (sh)RNA against CMTM6, either exclusively or in combination with shRNA against CMTM4, and found out that while the absence of CMTM6 decreased PD-L1, CMTM4 could attenuate this effect 26. Similar results were obtained in another NSCLC cell line, H2122. Moreover, Burr *et al.* carried out RNA interference and CRISPR-Cas0 knockout of CMTM6 in the lung adenocarcinoma cell line, HCC-827, resulting in the reduction of PD-L1 59. A Protein Atlas search of the relationship between *CMTM6* mRNA expression and prognosis was consistent with these findings (Figure 3).

### CMTM7 and NSCLC

#### Features of CMTM7 and expressed products

**Figure 4:**
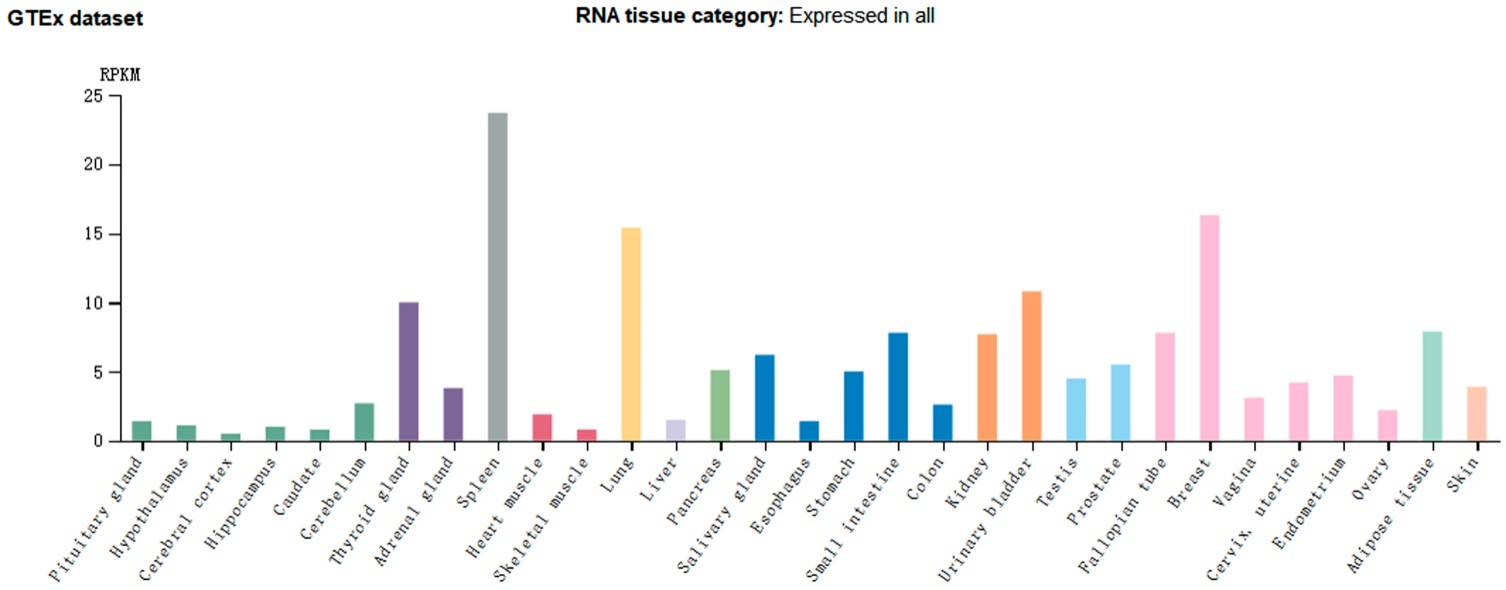
*CMTM7* mRNA expression is relatively higher in tissues from immune, hematopoietic, lung, and reproductive organs. (Image credit: Human Protein Atlas)

*CMTM7* also belongs to the CMTM superfamily, is located on chromosome 3p22.3 between *CMTM6* and *CMTM8* and within the same cluster, and is 63858 bp in length. It is highly expressed in leucocytes and has six splicing isoforms: CMTM7-001 to CMTM7-006. Of these, CMTM7-003 is not translated because of its retained introns, while CMTM7-004 and CMTM7-006 are not translated because of nonsense-mediated mRNA decay. The main splicing isoform (CMTM7-001) can be detected by northern blotting; its cDNA is 1369 bp long, including four introns, five exons, a classical promoter sequence, and a poly(A) tail 60. *CMTM7* mRNA expression in healthy human tissues shows organ specificity, with significantly higher expression observed in the spleen, breast, and lung (Figure 4).

#### CMTM7 is associated with the survival of lung cancer patients

**Figure 5:**
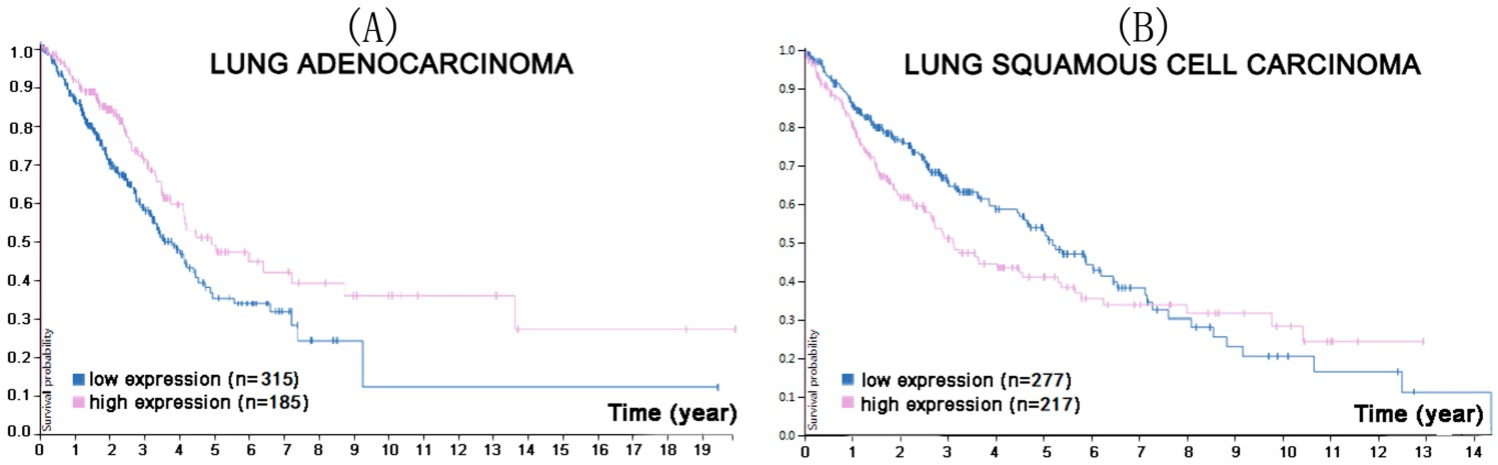
In the course of lung adenocarcinoma (A) and lung squamous cell carcinoma (B) progression, lower CMTM7 expression was associated with a lower survival probability for lung adenocarcinoma (p<0.0066), but a higher survival probability for lung squamous cell carcinoma (p<0.058). (Image credit: Human Protein Atlas. https://www.proteinatlas.org/ENSG00000153551-CMTM7/pathology/tissue/lung+cancer)

Liu 43 reported that CMTM7 was mainly expressed in the nuclei of tumor tissues of 127 lung adenocarcinoma patients, but in the cytoplasm and cell membrane of adjacent normal tissues. Total expression was either increased (43/127), decreased (54/127), or remained unchanged (30/127) in these patients, with abnormal expression always associated with a lower survival rate 43. This indicated an important role for CMTM7 in lung cancer. Figure 5 shows the connection between CMTM7 expression and patient survival probability.

CMTM7 mainly affects the EGFR-phosphoinositide 3-kinase (PI3K)/protein kinase B (AKT) pathway by upregulating the expression of p27 and downregulating cyclin-dependent kinase 2 (CDK2) and CDK6 to arrest the cell cycle at G1/S phase and slow cell growth 61. In cells transfected with CMTM7, more EGFR was transferred from the cell membrane to the cytoplasm and degraded than in control cells, and AKT showed decreased phosphorylation.

**Figure 6:**
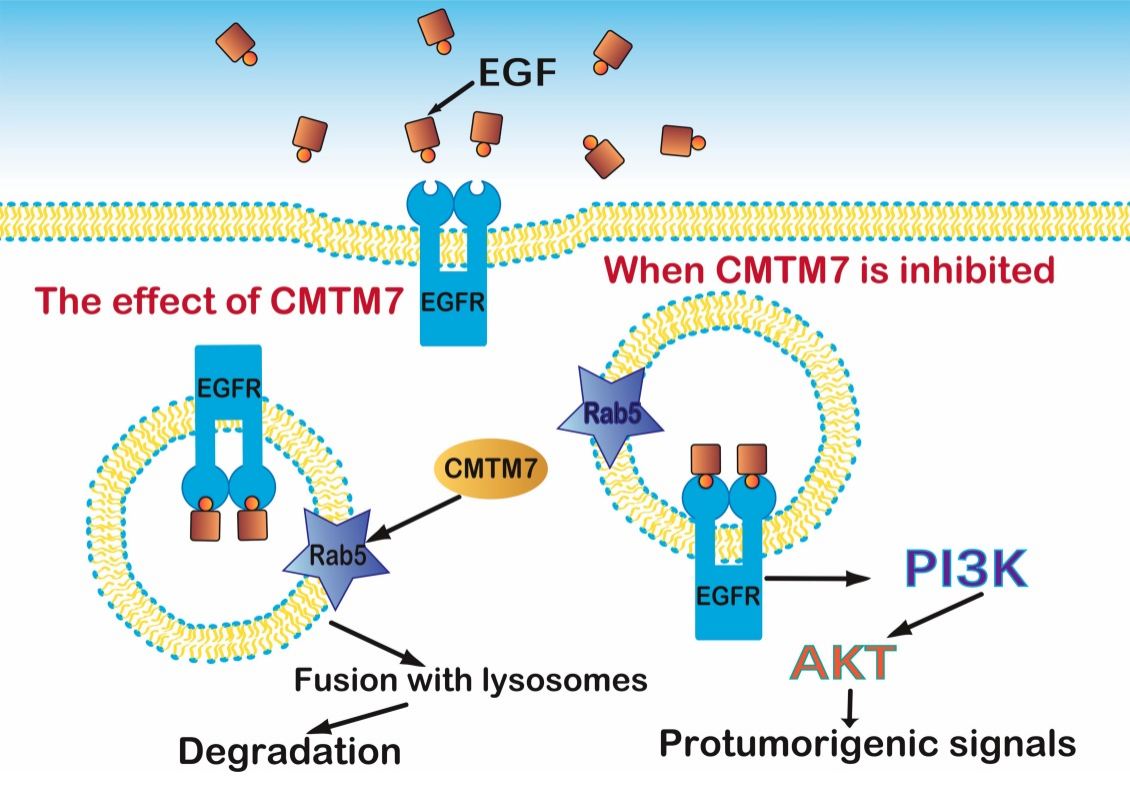
CMTM7 activates Rab5 which controls the fusion of early endosomes and affects EGFR protein trafficking, an essential part of the PI3K/AKT signaling pathway.

To further understand the molecular mechanism of CMTM7 within NSCLC, Liu 62 carried out *CMTM7* knockdown in A549 cells. They observed delayed degradation and increased internalization of EGFR, which are closely connected with the transportation and fusion of endosomes. *CMTM7* knockdown also decreased EGFR ubiquitination, which further delayed its degradation. Moreover, CMTM7 co-localized with Rab5 and EEA1 in early endosomes and aided endosome fusion. However, it inhibited the interaction of Rab5 and Rabaptin5, which is a critical step that affects fusion. That suggested that CMTM7 blocks the EGFR-AKT pathway by promoting EGFR internalization via endosomes (Figure 6).

Additional research by Liu 63 probed the effect of RNA interference in knocking down the expression of *CMTM7* in A549 cells. This showed that CMTM7 sustained and promoted the growth of tumor cells and inhibited apoptosis, while flow cytometry detected relatively more cells in G0/G1 phase than in S or G2/M phase. Together, these findings indicated that CMTM7 inhibits apoptosis in a “tumor promoting” mechanism through influencing the cell cycle. However, the contradiction of this finding with other studies and the unclear mechanism of action reflect the complexity of the relationship between CMTM7 and lung cancer, suggesting a need for further research.

### CMTM8 and NSCLC

**Figure 7:**
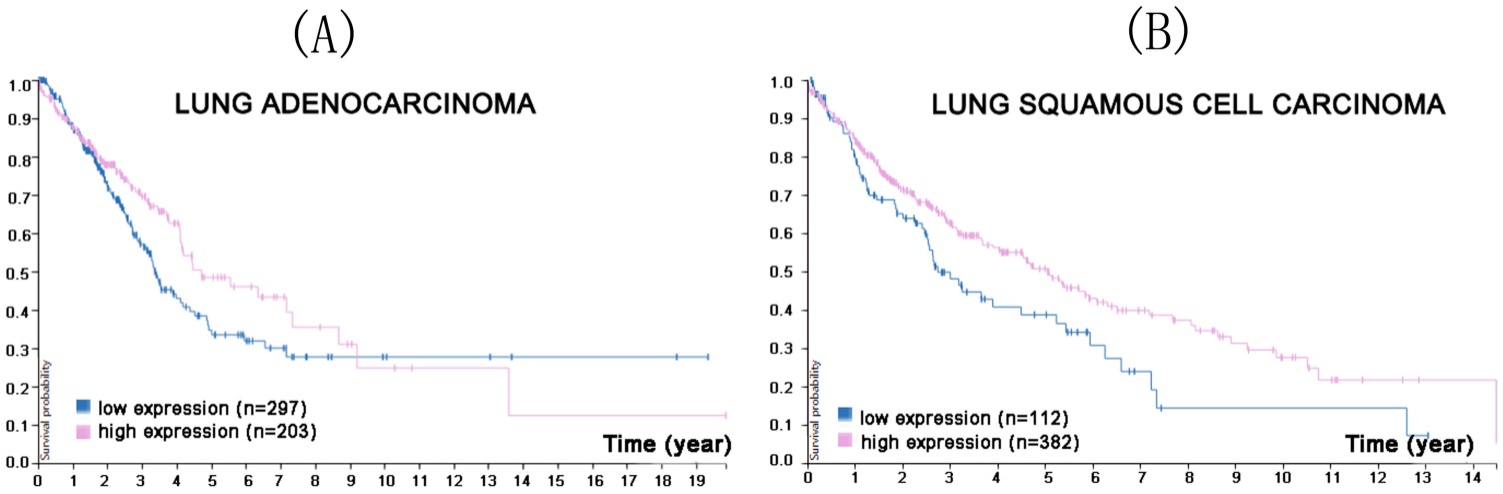
In the course of lung adenocarcinoma (A) and lung squamous cell carcinoma (B) progression, lower CMTM8 expression was associated with a lower survival probability (p<0.099 for lung adenocarcinoma, p<0.018 for lung squamous cell carcinoma). (Image credit: Human Protein Atlas. https://www.proteinatlas.org/ENSG00000170293-CMTM8/pathology/tissue/lung+cancer)

*CMTM8* is located on chromosome 3p23 and its full length of cDNA is 1185bp, of which nucleotides 295-816 encode CMTM8 8. The expression product is a four-time transmembrane protein consisting of 173 amino acids and MARVEL domains for vesicular transport and membrane ligation 8. CMTM8 is widely expressed in many normal human tissues and is frequently downregulated or absent in multiple solid tumors (liver, lung, colon, rectum, esophagus, and stomach) 13, 36, 37, 64, 65. CMTM8 inhibition promotes tumor cell proliferation, migration and invasion 8. Although the anti-tumor mechanism of CMTM8 is unclear, it is thought to accelerate the internalization of transferrin receptor and EGFR, and to be involved in a number of signal pathways associated with tumorigenesis and development 8, 14, 15. To elucidate further details, it is important to study the relationship between CMTM8 and a diverse range of tumor with the aim of identifying new targets of tumor gene therapy 13. A Protein Atlas search found that reduced CMTM8 expression was associated with a lower survival probability in lung squamous cell carcinoma progression (Figure 7).

## Conclusions and Prospects

Lung cancer is one of the most common cancers, with a reported dramatic rise in mortality and morbidity. Research into the relationship of the potential tumor suppressor gene *CMTM7* with lung cancer has so far focused on NSCLC, and identified a complex association that could exert either a positive or negative effect on tumor cells. CMTM research is still in its infancy, but investigations of clinical samples, patient prognostic data, *in vitro* and *in vivo* models, signal transduction pathways, and protein interactions provide a greater comprehension. Understanding the biological function and mechanism of CMTM in tumors will help provide a theoretical and experimental basis for effective tumor diagnosis, prognostic assessment, and improved sensitivity in radiotherapy, chemotherapy, and targeted therapy.
